# Affective Valence Regulates Associative Competition in Pavlovian Conditioning

**DOI:** 10.3389/fnbeh.2022.801474

**Published:** 2022-03-11

**Authors:** Vincent Laurent, R. Frederick Westbrook, Bernard W. Balleine

**Affiliations:** School of Psychology, The University of New South Wales, Sydney, NSW, Australia

**Keywords:** Pavlovian conditioning, incentive learning, appetitive-aversive interactions, prediction error, affect, valence, motivation

## Abstract

Evidence suggests that, in Pavlovian conditioning, associations form between conditioned stimuli and multiple components of the unconditioned stimulus (US). It is common, for example, to regard USs as composed of sensory and affective components, the latter being either appetitive (e.g., food or water) or aversive (e.g., shock or illness) and, therefore, to suppose different USs of the same affective class activate a common affective system. Furthermore, evidence is growing for the suggestion that, in competitive learning situations, competition between predictive stimuli is primarily for association with the affective system activated by the US. Thus, a conditioned stimulus (CS) previously paired with one US will block conditioning to another CS when both are presented together and paired with a different US of the same affective class, a phenomenon called transreinforcer blocking. Importantly, similar effects have been reported when steps are taken to turn the pretrained CS into a conditioned inhibitor, which activates the opposing affective state to the excitor that it inhibits. Thus, an appetitive inhibitor can block conditioning to a second CS when they are presented together and paired with foot shock. Here we show that the same is true of an aversive inhibitor. In two experiments conducted in rats, we found evidence that an aversive inhibitor blocked conditioning to a second CS when presented in a compound and paired with food. Such findings demonstrate that affective processes and their opponency organize appetitive-aversive interactions and establish the valences on which they are based, consistent with incentive theories of Pavlovian conditioning.

## Introduction

**Figure 1:**
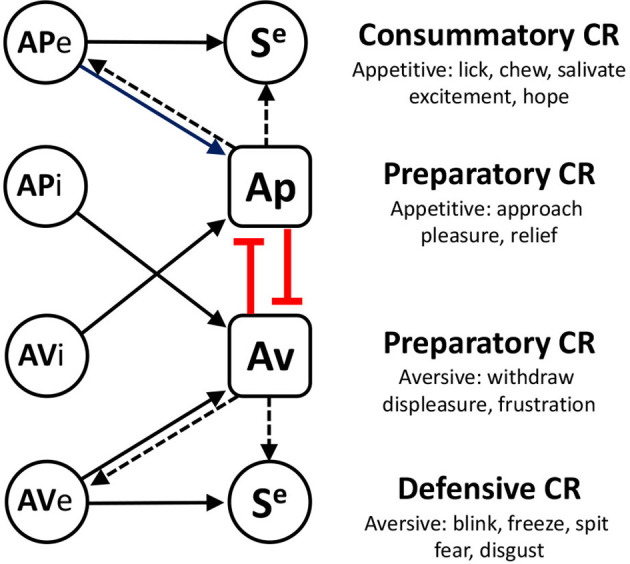
A summary of Konorski’s theory of Pavlovian incentive learning. Appetitive (APe) and aversive (AVe) excitors activate sensory specific (S^e^) and affectively specific (Ap or Av) components of the US representation to generate consummatory (defensive) or preparatory conditioned responses (CR), respectively. At the heart of this position is an opponency between appetitive and aversive affective systems which are assumed mutually to inhibit one another. One consequence of this view is that it provides a principled account of conditioned inhibition. Thus, appetitive inhibitors (APi) are aversive and so activate the aversive system whereas aversive inhibitors (AVi) are appetitive and activate the appetitive system. As a consequence, inhibitors can influence their concomitant excitors *via* this mutual inhibitory process (shown in red), reducing preparatory CR’s directly, and consummatory (and defensive) CR’s indirectly by reducing affective activation (dashed arrows).

One consequence of pairing a neutral cue with an unconditioned stimulus is that the former can take on some of the motivational and affective properties of the latter through a process of Pavlovian incentive learning. Perhaps the most sophisticated account of Pavlovian incentive learning is that developed by Konorski (1967)—see Figure 1. In this view Pavlovian conditioning comes in two forms: *consummatory* and *preparatory*; the former driven by associations between a conditioned stimulus (CS) and the sensory properties of the unconditioned stimulus(US), producing discrete conditioned responses (CRs) such as chewing or blinking (Debold et al., 1965; Schmajuk and Christiansen, 1990), and the latter by associations with the affective properties of the US, producing responses characteristic of the affective class to which that US belongs; approach when the US is appetitive and withdrawal when aversive (Dickinson and Dearing, 1979; Dickinson and Balleine, 2002). Although there is no doubting the importance of associations with the sensory properties of the US, the current research was focused on evaluating the nature of associations with the affective properties of the US.

In this context, the general claim, following Konorski, is that different USs from the same affective class activate a common affective system, perhaps the strongest evidence for which comes from studies of transreinforcer blocking. Blocking refers to the observation that a CS previously associated with a US can reduce, or block, conditioning to another CS when they are subsequently presented in compound and paired with the US (Kamin, 1968). However, whereas blocking typically employs the same US during initial and compound training, Bakal et al. (1974) observed that a CS pretrained with a foot shock US could block conditioning to a second CS when the compound was paired with a startle-eliciting auditory US, even though the sensory properties of these USs differ substantially. What they have in common, of course, is that they are aversive, and so transreinforcer blocking is usually taken as evidence that blocking is driven by competition for association with the affective system activated by the US. Transreinforcer blocking has also been observed using distinct appetitive USs; e.g., food pellets and sucrose solution for hungry rats (Rescorla, 1999) and food pellets and water for hungry and thirsty rats (Ganesan and Pearce, 1988).

Konorski also claimed that appetitive and aversive systems mutually inhibit one another, a claim supported by studies of counterconditioning in which pairing a CS with a mild paraorbital shock in rabbits was found to inhibit a previously established appetitive CRs to that CS (Lovibond and Dickinson, 1982) and *vice versa* (Scavio and Gormezano, 1980). Although such findings were important, perhaps even more important was the recognition that Konorski’s account of this interaction between contrasting motivational systems also provides a principled explanation for both the associative and affective properties of conditioned inhibitors. A conditioned inhibitor is a stimulus that signals the omission of an otherwise predicted US. From an affective perspective, appetitive inhibitors—that signal the omission of USs like food—are aversive whereas aversive inhibitors—that signal the omission of USs like a shock—are clearly appetitive. Therefore, if competition in Pavlovian conditioning is for association with the affective properties of the US, an appetitive inhibitor should block conditioning to an aversive excitor and an aversive inhibitor should block conditioning to an appetitive excitor—see Figure 1.

The original findings supporting this claim were reported in a chapter some years ago (Dickinson and Dearing, 1979). More recently, we have been able to replicate one part of this report, finding evidence that a CS that predicts the omission of a food US blocks aversive conditioning of its associate across parings of the compound and foot shock (Laurent et al., 2018). The current experiments investigated the opposite arrangement, evaluating the capacity of an aversive inhibitor to block appetitive conditioning to its associate across pairings of the compound and a food US. The aversive inhibitory CS was generated using backward conditioning during which the stimulus was presented a few seconds after the administration of a foot shock US. To confirm the efficacy of this arrangement, an aversive excitory CS was also established through standard forward conditioning during which the stimulus immediately preceded the administration of the foot shock US. Experiment 1 investigated the effect of the backward CS-shock pairings (i.e., shock-S2) against forward pairings (i.e., S1-shock). Experiment 2 compared the influence of these forward and backwardly paired CSs against neutral control CSs.

## Methods

### Subjects

The subjects were 24 experimentally naive male Sprague-Dawley rats (Rattus Norvegicus; 8–12 weeks old; 300–500g) obtained from the Animal Resources Center (Perth, WA). They were housed in plastic boxes (67 cm × 40 cm × 22 cm; eight rats per box) located in a climate-controlled colony room. The room was maintained on a 12 h light/dark cycle (lights on between 7 a.m and 7 p.m) and all procedures took place during the light cycle. Three days before the behavioral procedures, the rats were handled daily and put on a food deprivation schedule to maintain them at around 90% of their free feeding weight. The Animal Care and Ethics Committee of the University of New South Wales approved all procedures.

### Behavioral Apparatus

Training and testing took place in 12 Med Associates (St. Albans, VT, USA) operant chambers enclosed in sound- and light-resistant shells. The floors consisted of stainless-steel rods that were 3.8 mm in diameter, spaced 1.6 cm apart (center to center), and wired to a constant current generator that could deliver a shock. Each operant chamber was equipped with a pellet dispenser that could deliver a single grain pellet (45 mg; BioServe Biotechnologies) into a recessed magazine, an auditory stimulus generator, that could deliver a 1 kHz tone stimulus, and a 28 V DC mechanical relay that generated a 2 Hz clicker stimulus. There were three stimulus lights, two positioned on the same wall to either side of the magazine, providing a 2 Hz flashing visual stimulus when activated, and a 3 W, 24 V house light positioned on the opposite wall. An infrared photobeam allowed for the detection of magazine entries and a camera mounted on the back wall of each shell and connected to a monitor and a DVD recorder located in another room, recorded the behavior of each rat. An infrared light source illuminating each chamber was used to visualize behavior conducted in the dark (i.e., the house light was used as a discrete stimulus). A set of two microcomputers running proprietary software (Med-PC; MED Associates) controlled all experimental events and recorded magazine entries.

### Behavioral Procedures

#### General Procedures

Four distinct stimuli were used (S1, S2, S3, and S4): clicker, tone, the constant house light, and flashing stimulus lights. The duration of the individual stimuli or compounds of these stimuli (always one visual and the other auditory) was 20 s. In any single session, presentations of the stimuli or compounds were separated by an inter-trial interval that ranged from 4 min to 6 min with an average of 5 min. In all experiments, the intensity of the foot shock was 0.5 mA with a duration of 0.5 s.

All experiments started with two sessions of pre-exposure across two consecutive days during which each stimulus was presented twice. Two sessions of magazine training were then given across two consecutive days for 30 min during which food pellets were delivered on random-time 60 s schedule. The aim of these sessions was to familiarize the rats with the pellets and overcome neophobia.

#### Experiment 1

*Aversive training—*Following pre-exposure and magazine training, two sessions of aversive training were given each day for six consecutive days. One session involved forward conditioning in which four presentations of S1 terminated in the delivery of foot shock. The other session involved backward conditioning in which each of the four deliveries of the foot shock were followed 5 s later by presentation of S2. The order of the sessions was counterbalanced across days. For half of the rats, S1 and S2 were auditory (tone and clicker, counterbalanced) whereas for the remainder they were visual (constant house light or flashing stimulus lights, counter balanced). Aversive responses (freezing) were recorded across conditioning in the presence of S1 and S2 and the 20 s period preceding (pre) either S1 or the shock in the case of the S2 sessions.

*Appetitive training*—The day after aversive training, rats received an additional magazine training session in the manner described. Next, appetitive training was given across two sessions on two consecutive days. In each session, S1 was presented in compound with S3 and S2 in compound with S4. Presentations of the S1S3 and the S2S4 compounds terminated in the delivery of a food pellet. All compounds were pseudo-randomly presented four times in each session. The stimuli were counterbalanced such that S3 and S4 were auditory in rats for which S1 and S2 were visual, whereas S3 and S4 were visual in rats for which S1 and S2 were auditory. Only two sessions of appetitive training were administered to avoid extinction of the aversive properties of the aversive CSs. Throughout these sessions, magazine entries were recorded and separated into stimulus and compound periods and pre-stimulus and pre-compound periods of equal length (20 s).

*Appetitive testing—*A single test session was administered 24 h after the end of appetitive training. The two previously trained compounds (S1S3 and S2S4) were presented once followed by the delivery of the food pellet outcome. Then, stimuli S3 and S4 were presented alone twice in the following order: S3-S4-S4-S3. Magazine entries were recorded and separated into stimulus/compound period and a pre-stimulus/compound period with equal length (20 s).

#### Experiment 2

*Aversive training—*Following pre-exposure and magazine training, eight consecutive days of aversive training were administered. There was a single daily session across the first 4 days. For half of the rats (Group Forward), the session involved forward conditioning to stimulus S1 in the manner described previously. For the other half of the rats (Group Backward), the session consisted of backward conditioning to stimulus S1 in the manner described previously. From day 5 of aversive training, the rats received an additional training session each day for the next 4 days during which a second stimulus S2 was presented without any consequence. The two daily sessions (the one with S1 and the one with S2) were given at least 2 h apart and the order in which they occurred was counterbalanced. For half of the rats in each group, S1 and S2 were visual stimuli (constant house light or flashing stimulus lights, counterbalanced), whereas they were auditory stimuli (clicker or tone, counterbalanced) for the other half. Aversive responses were recorded during the 20 s stimulus period and the immediately preceding 20 s period (pre) in the group that received forward conditioning and during the stimulus period and the 20 s period immediately prior to the delivery of the foot shock (pre) in the group that received backward conditioning.

Aversive training was followed by one session of magazine training, two daily sessions of appetitive training and one session of appetitive testing. These sessions were identical to those described in Experiment 1.

### Statistical Analyses

Appetitive responding was recorded by the Med Associates software. Aversive responding was rated in a time-sampling manner and judged as either freezing or not freezing every 2 s by a trained observer blind to the subjects’ group assignment. A proportion of test data was cross-scored by a second naïve observer; there was a high level of agreement between observers (Pearson product moment correlation >0.9). Freezing was defined as the absence of all movement, except those related to breathing. The differences between groups or stimuli were analyzed by means of planned orthogonal contrasts. Within-session changes in responding were assessed by a planned linear trend analysis. All these procedures and analyses have been described by Hays (1973); see also Harris (1994) and were conducted in the PSY software (School of Psychology, The University of New South Wales, Australia). The Type I error rate was controlled at α = 0.05 for each contrast tested. If interactions were detected, follow-up simple effects analyses were calculated to determine the source of the interaction.

## Results

### Experiment 1

**Figure 2:**
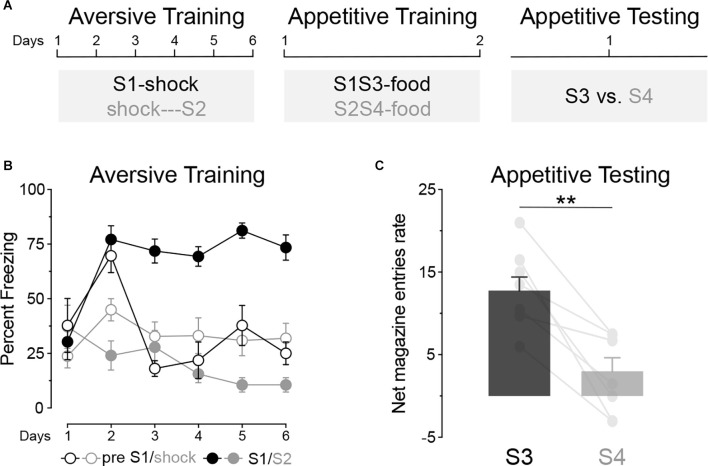
The opposing effect of an aversive excitor and an aversive inhibitor on appetitive conditioning. **(A)** Design of Experiment 1; S1/S2/S3/S4: clicker, tone, flashing light or constant light (counterbalanced). **(B)** Aversive conditioning: S1 became an aversive excitor whereas S2 became into an aversive inhibitor. **(C)** Appetitive conditioning test: stimulus S3 elicited significantly more appetitive responding than stimulus S4 at test. Error bars denote ± 1 SEM. Asterisks denote significant effect (**p < 0.01).

Experiment 1 used a within-subjects design to examine whether an aversive excitor and an aversive inhibitor exert distinct effects on appetitive conditioning. The design, shown in Figure 2A, had three stages: aversive conditioning, in which rats (*n* = 8) received S1 paired forwardly and S2 paired backwardly with foot shock, preceding S2 by a few seconds (Moscovitch and LoLordo, 1968); appetitive conditioning, in which S1 and S2 were presented in compound with two novel stimuli, S3 and S4, to form S1S3 and S2S4 compounds paired with a food pellet outcome; and a test phase in appetitive conditioning to S3 and S4 was assessed.

#### Training

During aversive training (Figure 2B), the forwardly paired S1 elicited more freezing than the backwardly paired S2 (Period; S1 vs. S2; *F*_(1, 7)_ = 107.24, *p* < 0.001) and this difference grew larger as training progressed (Period × Days; *F*_(1, 7)_ = 17.19, *p* < 0.01). Freezing in the absence of the stimuli (the “pre” period) was similar in the forward and backward sessions (pre S1 vs. pre shock; *F* < 0.5). Pre-responding was lower than that to S1 (pre vs. S1; *F*_(1, 7)_ = 51.63, *p* < 0.001), confirming that fear was increased by S1. By contrast, pre-responding was greater than that elicited by S2 (pre vs. S2; *F*_(1, 7)_ = 7.894, *p* < 0.05), confirming that fear was reduced by S2. These results suggest, therefore, that S1 became an aversive excitor and S2 an aversive inhibitor. During appetitive training (Table 1) no significant difference was found between the S1S3 and S2S4 compounds or in responding in the presence or absence of these compounds (*F*s < 1.3).

**Table 1:** Appetitive training data for Experiment 1—Magazine entries per minute (mean ± s.e.m) in the absence (pre) or presence of the two compounds S1S3 and S2S4.

Days	Magazine entries per minute ± (mean s.e.m)		
	pre	S1S3	S2S4
1	5.86 ± 1.05	5.53 ± 1.66	5.25 ± 1.23
2	6.61 ± 0.65	7.87 ± 1.86	7.13 ± 1.13

#### Test

The data from the final test are shown in Figure 2C. It is clear that stimulus S3 elicited more appetitive responding than stimulus S4 (S3 vs. S4; *F*_(1, 7)_ = 21.90, *p* < 0.01) suggesting that the aversive inhibitor, S2, attenuated conditioning to the neutral S4 stimulus.

### Experiment 2

**Figure 3:**
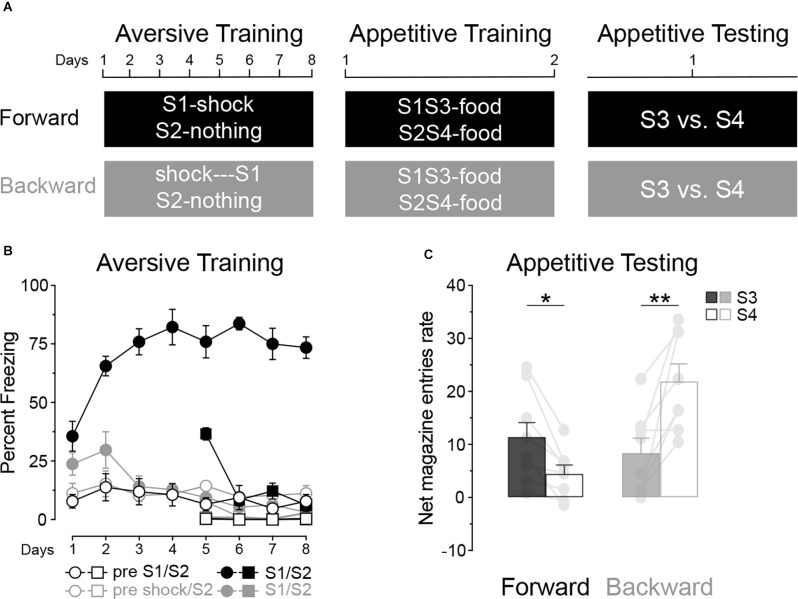
An aversive excitor enhances, whereas an aversive inhibitor blocks, appetitive conditioning. **(A)** Design of Experiment 2; S1/S2/S3/S4: clicker, tone, flashing light or constant light (counterbalanced). **(B)** Aversive conditioning: S1 became an aversive excitor in group Forward and an aversive inhibitor in group Backward whereas S2 was neutral in both groups. **(C)** In group Forward, stimulus S3 elicited more appetitive responding than stimulus S4. The opposite was found in group Backward. Error bars denote ± 1 SEM. Asterisks denote significant effect (*p < 0.05; **p < 0.01).

Although consistent with the claim that the aversive inhibitor, S2 in Experiment 1, blocked appetitive conditioning to the added stimulus, S4, it is possible that the aversive excitor, S1, increased conditioning to the neutral stimulus S3 and, as it stands, it is not possible to establish whether conditioning was elevated to S3 or was depressed to S4. Experiment 2 sought to resolve this issue using the between-subject design shown in Figure 3A. In the first aversive conditioning stage, two groups were used, Group Forward (*n* = 8) received forward pairings of S1 and foot shock, whereas Group Backward (*n* = 8) received backward pairings of S1 and shock. For both groups a second stimulus, S2, was presented alone unpaired with shock. In appetitive conditioning the two pre-trained stimuli in compound with two distinct novel stimuli, S1S3 and S2S4 and both were paired with the delivery of a food pellet. Finally, appetitive conditioning to S3 and S4 was assessed in a final test stage. As a consequence, in both groups S4 was compounded with a neutral stimulus, S2, which acted as a control to evaluate the influence of an aversive excitor (S1 in Group Forward) or an aversive inhibitor (S1 in Group Backward) on appetitive conditioning to S3.

#### Training

For aversive training (Figure 3B) we first considered performance to S1. This training revealed a significant difference in freezing between the two groups of rats, Group Forward and Backward (Group: Forward vs. Backward; *F*_(1, 14)_ = 119.85, *p* < 0.001), freezing in the presence vs. absence of S1 (Period: pre vs. S1; *F*_(1, 14)_ = 147.17, *p* < 0.001) and in freezing across the course of training (Group × Days; *F*_(1, 14)_ = 15.95, *p* < 0.01; and Period × Days; *F*_(1, 14)_ = 19.87, *p* < 0.001). Group Forward displayed more freezing in the presence of S1 than in its absence (Period: S1 vs. pre; *F*_(1, 7)_ = 220.07, *p* < 0.001) and this difference grew larger across training (Period × Days; *F*_(1, 7)_ = 16.22, *p* < 0.01). By contrast, Group Backward quickly reduced responding to S1 (Days; *F*_(1, 7)_ = 5.82, *p* < 0.05) such that freezing was similar in the presence and absence of S1 (Period: pre vs. S1; *F* < 0.3). These patterns of performance remained unaffected by the introduction of the S2 sessions on days 5–8. Overall, S2 elicited less aversive responding than S1 (Period 1: S2 vs. S1; *F*_(1, 14)_ = 164.79, *p* < 0.001) but more than when the stimuli were absent (Period 2: S2 vs. pre; *F*_(1, 14)_ = 14.56, *p* < 0.01). Yet, these differences depended on the group of rats considered (Period 1 × Days; *F*_(1, 14)_ = 120.59, *p* < 0.001; Period 2 × Days; *F*_(1, 14)_ = 103.06, *p* < 0.001). In group Forward, S2 elicited less aversive responding than S1 (S2 vs. S1; *F*_(1, 4)_ = 154.74, *p* < 0.001) but more than when the stimuli were absent (S2 vs. pre; *F*_(1, 7)_ = 277.99, *p* < 0.001). By contrast, in Group Backward, S2 elicited more aversive responding than S1 (S2 vs. S1; *F*_(1, 7)_ = 10.32, *p* < 0.05) and more in the presence than in the absence of the stimuli (S2 vs. pre; *F*_(1, 7)_ = 14.99, *p* < 0.01). Taken together, these results are consistent with the view that S1 became an aversive excitor in Group Forward and an aversive inhibitor in Group Backward, whereas S2 was neutral in both groups. In appetitive conditioning (Table 2) no significant differences were found between groups, compounds or responding in the presence or absence of these compounds (all *F*s < 3.9).

**Table 2:** Appetitive training data for Experiment 2—Magazine entries per minute (mean ± s.e.m) in the absence (pre) or presence of the two compounds S1S3 and S2S4.

Days	Groups	Magazine entries per minute ± (mean s.e.m)		
		pre	S1S3	S2S4
1	Forward	8.30 ± 1.83	7.66 ± 0.87	6.75 ± 2.39
	Backward	11.20 ± 1.68	10.78 ± 2.22	7.97 ± 2.04
2	Forward	6.38 ± 1.77	10.84 ± 1.89	11.62 ± 3.08
	Backward	9.23 ± 1.89	14.5 ± 3.66	12.72 ± 3.04

#### Test

The data from the final test are presented in Figure 3C. Appetitive responding to S3 relative to S4 depended on group (Groups × Period; *F*_(1, 14)_ = 26.80, *p* < 0.001). Thus, S3 elicited more responding than S4 in Group Forward (Period: S3 vs, S4; *F*_(1, 7)_ = 12.24, *p* < 0.05) whereas it elicited less responding than stimulus S4 in Group Backward (Period: S3 vs, S4; *F*_(1, 7)_ = 15.64, *p* < 0.01). These test data reveal, therefore, that, whereas an aversive inhibitor can block appetitive conditioning to a neutral CS, an aversive excitor can enhance appetitive conditioning when both are presented together and paired with food. This experiment suggests, therefore, that the effect in Experiment 1 was induced by both a facilitation of conditioning to S3 and by blocking conditioning to S4.

## Discussion

The results of the current experiments have four important implications for theories of Pavlovian conditioning generally and appetitive-aversive interactions in particular. The first is the most obvious; the current findings replicate those reported in the Dickinson and Dearing (1979) chapter, that aversive inhibitors and appetitive excitors have similar properties, and so support Konorski’s view of Pavlovian incentive learning. Here we established that backwardly pairing a shock with a discrete CS is sufficient to allow that CS to compete with another CS for association with the appetitive affective system when both were presented in a compound and paired with food. In Experiment 1 this reduction was relative to a stimulus compounded with an aversive excitor and, therefore, we could not definitively establish whether the effect reflected a reduction in the former or an elevation in the latter. This was clarified in Experiment 2 using a between-subjects design, with the effect of the aversive inhibitor vs. the aversive excitor compared against a control CS. There it was established less ambiguously that the aversive inhibitor blocked conditioning to the added CS when presented in a compound and paired with food. Along with other examples of transreinforcer blocking, therefore, these results confirm that competitive learning processes in Pavlovian conditioning are best viewed as competing for association with the affective processes activated by the US, whether that competition is generated directly within affective systems or indirectly *via* their interaction.

### Context Predictions

Evidence indicates that, as backward conditioning imbues a CS with inhibitory properties, it can also confer excitatory properties to the background context (Chang et al., 2003). This raises the possibility that the expectation of shock in the context influenced conditioning to the novel CSs across appetitive training. To minimize this possibility, our experiments included magazine training sessions before and immediately after aversive training to reduce the aversive properties of the context. The lack of differences in responding in the absence of the stimuli during appetitive training (Tables s 1 and 2) indicates that these sessions successfully reduced context fear. More importantly, the within-subjects design employed in Experiment 1 ensured that the expectation of shock in the context was similar during presentations of the backwardly- and forwardly-trained stimuli with their associates during appetitive training, and the two stimuli influenced appetitive conditioning to their associates in an opposite manner. We are therefore confident that the effects reported here were due to the predictive properties of the aversive stimuli rather than those of the context.

### Appetitive-Aversive Predictions and Prediction Errors

**Figure 4:**
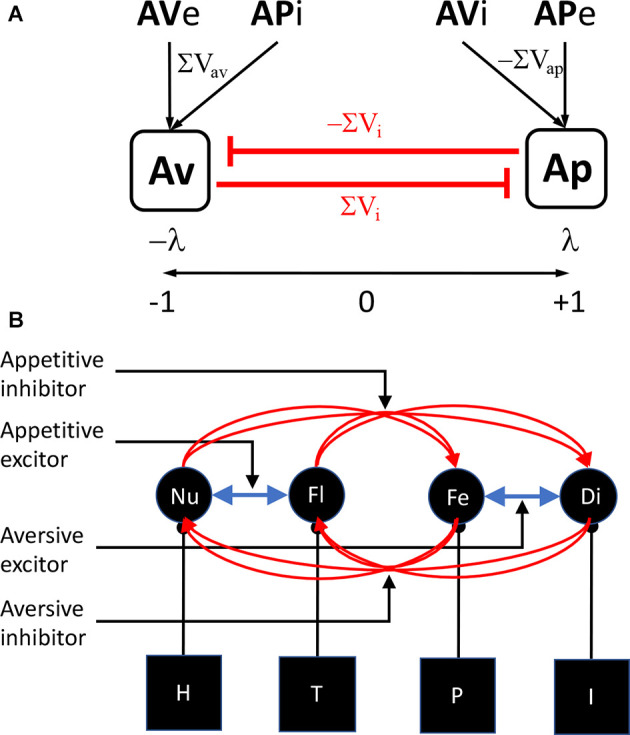
Models of appetitive-aversive interaction. **(A)** An appetitive-aversive continuum account according to which appetitive (λ) and aversive (−λ) events are combined on a substrate to generate the prediction error term for appetitive-aversive interactions. The associative strength of aversive excitors and appetitive inhibitors (AVe+APi) sum (ΣV_av_) to drive aversive predictions, aversive prediction errors (−λ+ΣV_av_) and inhibition of the appetitive system (ΣVi) whereas the associative strength of appetitive excitors and aversive inhibitors (APe+AVi) sum (ΣV_ap_) to drive appetitive predictions, aversive prediction errors (λ−ΣV_ap_) and inhibition of the aversive system (−ΣVi). **(B)** A motivational account on which specific motivational systems combine to emulate appetitive and aversive systems and drive inhibitory connections with systems to which they are not linked. In this example, a hunger (H)—nutrient (Nu) system joins a thirst (T)—fluid (Fl) system to generate appetitive activity and a pain (P)—fear (Fe) system joins an illness (I)—disgust (Di) system to generate aversive activity (blue arrows) and these pairs of systems maintain individual inhibitory links (red arrows) to generate appetitive-aversive interactions.

Experiment 2 also provided evidence for another phenomenon dependent on appetitive-aversive interaction: appetitive superconditioning. Dickinson (1977) reported that, when a novel CS was presented with a CS previously paired with food and the compound paired with shock, conditioning to the novel CS was enhanced relative to a control stimulus shocked in a compound with a CS unpaired with food. Here we found evidence that a novel CS presented in a compound with an aversive inhibitor and paired with food showed enhanced appetitive conditioning, again compared to a stimulus paired with food in a compound with CS unpaired with shock. Superconditioning is in many ways the opposite of blocking and is perhaps best interpreted as driven by the increased discrepancy between the predicted outcome, shock in the case of the S1S3 compound in Group Forward of Experiment 2, and the food that was actually delivered. Nevertheless, both the superconditioning induced by this enhanced prediction error in Group Forward and the transreinforcer blocking induced by the reduced prediction error in Group Backward present a problem. These explanations not only depend on the interaction between appetitive-aversive affective systems, they also depend on a common prediction error signal derived from that interaction. The question is how does that common prediction error arise? Although this issue has not been directly addressed in the current literature, these phenomena could be argued to suggest the existence of a substrate that establishes an appetitive-to-aversive continuum on which, for example, appetitive USs and CSs generate positively signed predictions and aversive USs and CSs negatively signed predictions, with predictions from appetitive and aversive inhibitors signed oppositely to their excitors (Figure 4A). Such a substrate would provide a straightforward basis for generating the net affective prediction necessary to calculate a common prediction error.

The alternative is that there exists a range of more specific motivational control processes that are independent but can interact in a manner able to emulate the activity of distinct affective systems (Figure 4B); e.g., excitatory interactions between pain and illness systems, productive of fear and disgust, could emulate general aversive activity whereas interactions between nutrient and fluidic predictions, driven by hunger and thirst systems, could emulate general appetitive activity, a suggestion for which there is some evidence (Balleine and Dickinson, 2006). In contrast, inhibitory interactions between such systems, or perhaps a subset of them, could subserve conditioned inhibition, with their net effects resulting in general excitatory and inhibitory predictions and prediction errors. There is, at present, little evidence on which decide between these accounts although, in a recent study, we found some evidence for the latter in showing that shifts in primary motivational state, for example a shift from hunger to satiety, reduced the impact of the prediction error on aversive superconditioning to a neutral CS when a compound composed of that CS and an appetitive excitor was paired with foot shock (Laurent et al., 2018; see also Balleine and Dickinson, 2006).

### The Importance of Appetitive-Aversive Interactions

Generally, therefore, the current data, when combined with previous findings, suggest that experiments investigating the factors controlling appetitive-aversive interactions may provide an interesting test bed for theories of Pavlovian conditioning. Such interactions are, of course, very common—few situations are entirely appetitive or entirely aversive—and although most associative theories can be applied equally to appetitive or aversive conditioning, these theories have yet to develop a systematic approach to the opponency between affective processes at the heart of their interaction. There have certainly been very prominent opponent process theories, mostly concerned with specifying when and under what conditions appetitive and aversive states arise and that were successfully applied to some behavioral phenomena—particularly those generated by addiction (Solomon and Corbit, 1974). Another approach was developed more formally into Wagner’s (1981) Sometimes Opponent Processes (SOP) model of conditioning and, indeed some aspects of Konorski’s theory of Pavlovian incentive learning permeated a later version of that model as an affective-emotional extension to SOP (i.e., AESOP; Wagner and Brandon, 1989). This later version explored the effects of partitioning the US into sensory and affective components but, unfortunately, did not extend the account to formally investigate how appetitive-aversive interactions might be considered or how excitatory and inhibitory processes of opposing valence interact.

## Data Availability Statement

The raw data supporting the conclusions of this article will be made available by the authors, without undue reservation.

## Ethics Statement

The animal study was reviewed and approved by The Animal Care and Ethics Committee of the University of New South Wales.

## Author Contributions

VL and RW designed the experiments. VL conducted the experiments and analyzed the data. VL and BB wrote the manuscript. All authors contributed to the article and approved the submitted version.

## Conflict of Interest

The authors declare that the research was conducted in the absence of any commercial or financial relationships that could be construed as a potential conflict of interest.

## Publisher’s Note

All claims expressed in this article are solely those of the authors and do not necessarily represent those of their affiliated organizations, or those of the publisher, the editors and the reviewers. Any product that may be evaluated in this article, or claim that may be made by its manufacturer, is not guaranteed or endorsed by the publisher.
